# Efficient Commitment to Functional CD34+ Progenitor Cells from Human Bone Marrow Mesenchymal Stem-Cell-Derived Induced Pluripotent Stem Cells

**DOI:** 10.1371/journal.pone.0034321

**Published:** 2012-04-09

**Authors:** Yulin Xu, Lizhen Liu, Lifei Zhang, Shan Fu, Yongxian Hu, Yingjia Wang, Huarui Fu, Kangni Wu, Haowen Xiao, Senquan Liu, Xiaohong Yu, Weiyan Zheng, Bo Feng, He Huang

**Affiliations:** 1 Bone Marrow Transplantation Center, First Affiliated Hospital, Zhejiang University School of Medicine, Hangzhou, Zhejiang Province, China; 2 School of Biomedical Sciences, The Chinese University of Hong Kong, Hong Kong, China; University of Medicine and Dentistry of New Jersey, United States of America

## Abstract

The efficient commitment of a specialized cell type from induced pluripotent stem cells (iPSCs) without contamination from unknown substances is crucial to their use in clinical applications. Here, we propose that CD34+ progenitor cells, which retain hematopoietic and endothelial cell potential, could be efficiently obtained from iPSCs derived from human bone marrow mesenchymal stem cells (hBMMSC-iPSCs) with defined factors. By treatment with a cocktail containing mesodermal, hematopoietic, and endothelial inducers (BMP4, SCF, and VEGF, respectively) for 5 days, hBMMSC-iPSCs expressed the mesodermal transcription factors Brachyury and GATA-2 at higher levels than untreated groups (*P*<0.05). After culturing with another hematopoietic and endothelial inducer cocktail, including SCF, Flt3L, VEGF and IL-3, for an additional 7–9 days, CD34+ progenitor cells, which were undetectable in the initial iPSC cultures, reached nearly 20% of the total culture. This was greater than the relative number of progenitor cells produced from human-skin-fibroblast-derived iPSCs (hFib-iPSCs) or from the spontaneous differentiation groups (*P*<0.05), as assessed by flow cytometry analysis. These induced cells expressed hematopoietic transcription factors TAL-1 and SCL. They developed into various hematopoietic colonies when exposed to semisolid media with hematopoietic cytokines such as EPO and G-CSF. Hematopoietic cell lineages were identified by phenotype analysis with Wright-Giemsa staining. The endothelial potential of the cells was also verified by the confirmation of the formation of vascular tube-like structures and the expression of endothelial-specific markers CD31 and VE-CADHERIN. Efficient induction of CD34+ progenitor cells, which retain hematopoietic and endothelial cell potential with defined factors, provides an opportunity to obtain patient-specific cells for iPSC therapy and a useful model for the study of the mechanisms of hematopoiesis and drug screening.

## Introduction

CD34+ progenitor cells are used to treat many disorders, such as incurable hematologic and lymphoid malignancies. However, immune incompatibility, or gene disparity between recipients and donors, causes graft-versus-host disease (GVHD) and other transplant-related complications. These can cause graft failure or even death [1]. Additionally, sources of transplantable CD34+ progenitor cells have been limited to bone marrow, umbilical cord blood, and mobilized peripheral blood tissues. This makes it difficult to obtain CD34+ progenitor cells in numbers sufficient to reconstitute a normal blood system for an adult [2], [3]. The high risk of fatal complications and the quantity requirements for CD34+ progenitor cells have prevented cell therapy from gaining wide application. The study and establishment of efficient CD34+ progenitor cell induction systems are thus necessary [4], [5].

Human embryonic stem cells (hESCs) are derived from the inner cell mass of preimplantation embryos. These cells are totipotent, capable of giving rise to all types of human cells and of proliferating indefinitely *in vitro* under specific culture conditions. Since the establishment of the first hESC line, people have been immensely interested in using embryonic stem cells as an alternative source of CD34+ progenitor cells for cell transplantation [6]–[13]. However, approaches using embryoid body (EB) formation or co-culture with a feeder layer of mouse cells, such as the OP9 stromal cell line or the S17 bone marrow cell line, are not suitable for clinical applications. This is not only because of the low differentiation efficiency, poorly defined induction components, and contamination from animal sources, but also because of the immune incompatibility and the ethical and legal restrictions surrounding embryonic stem cell research [7].

The emerging of induced pluripotent stem cells (iPSCs) was a breakthrough. Potentially patient-specific cells can be obtained without the ethical concerns or immune rejection. They represent a potentially unlimited source of functionally specific cell lineages [14]–[17]. iPSCs have been demonstrated to be a viable alternative to ESCs for generation of CD34+ progenitor cells, which is of immense importance to clinical treatment, drug discovery, and the study of disease mechanisms [10], [18]–[21]. Studies performed by Choi and his colleagues showed similar patterns of differentiation potential between human iPSC lines and hESC lines, as determined using an OP9 differentiation system [18]. Woods *et al.* developed an optimized differentiation protocol using mouse stromal cells with cytokines for the generation of hematopoietic lineages from iPSCs [19]. However, the cells obtained from the studies given above were contaminated with unidentified factors from the mouse-derived cells. This is not in line with the crucial step for cell-based therapy. Although several defined culture conditions have been identified for directing human iPSCs differentiation toward CD34+ progenitor cells, the overall efficacy of these protocols remains low [20], [21]. In one study, hiPSC-derived CD34+ cells cannot develop into hematopoietic cells [21]. Better approaches for more efficient induction of CD34+ progenitor cells merit investigation. Hematopoietic potential is strictly dependent on cellular signaling, as demonstrated by Kennedy *et al*., and existing protocols for the efficient induction of functional CD34+ progenitor cells appear to require treating the differentiating cells with complex cocktails of factors that affect the mesoderm, hematological, and endothelial cells [9], [22]–[25].

Adult human bone marrow-derived mesenchymal stem cells (hBMMSCs) are multipotent, meaning that they can differentiate into several lineages, such as osteoblasts, adipocytes, and chondrocytes [26]. They are closely related to hematopoiesis. hBMMSCs have immunosuppressive properties that make them interesting candidates for cell-based therapy in organ transplantation. They are currently used in regenerative medicine for tissue regeneration [27]. Although hBMMSCs can be easily expanded *in vitro*, they have limited lifespan and differentiation potentials in culture. This impedes further clinical application of these cells. However, hBMMSC-derived iPSCs (hBMMSC-iPSCs) may have greater potential in clinical applications with indefinite passages. They are also capable of forming three germ layers and are more immunologically compatible with patients than hBMMSCs are. They also have other advantages over other types of cell-derived iPSCs with respect to disease treatment [28]. Regarding their epigenetic state in hematopoiesis, we propose that hBMMSCs would be an excellent source of cells for the efficient generation of functional CD34+ progenitor cells.

In this study, based on the reprogramming of hBMMSCs to iPSCs by ectopic expression of four transcription factors OCT4, SOX2, c-MYC, and KLF4, we demonstrated that functional CD34+ progenitor cells retaining hematopoietic and endothelial cell potential could be obtained more efficiently from hBMMSC-iPSCs than from human-skin-fibroblast-derived iPSCs (hFib-iPSCs) by sequential exposure to two sets of defined chemicals and growth factors, which exert specific function on mesodermal, hematological and endothelial cell signaling pathways, including BMP4, SCF, Flt3L, VEGF, IL-3, and EPO. This induction system for CD34+ progenitor cells is not contaminated by unidentified substances because it does not involve mouse feeder layers or other poorly defined induction components, providing an opportunity to generate an unlimited number of human-leukocyte-antigen matched (HLA-matched) transplantable cells and constitutes an ideal cell model for the study of the mechanisms of hematopoiesis and related diseases and drug screening.

## Results

### Generation and characterization of hBMMSC-iPSCs

**Figure 1:**
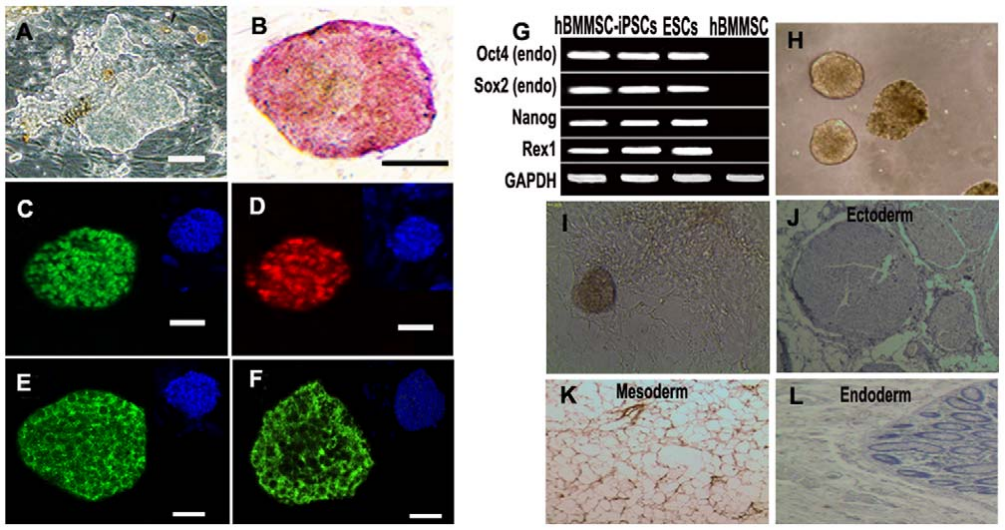
Generation and characterization of iPSCs from hBMMSCs. Representative hBMMSC-iPSCs colonies (A) and AP staining (B). (C-F) Immunoassay of hBMMSC-iPSC colonies expressing ESC specific markers as OCT4 (C), NANOG (D), TRA-1-81 (E) and SSEA-3 (F). 4, 6-Diamidino-2-phenylindole (DAPI) staining was used to reveal the nuclei. Scale bar, 100 µm. (G) RT-PCR analysis of endogenous pluripotent gene mRNA expression in hBMMSC-iPSCs. Total RNA was isolated from hBMMSC-iPSCs, ESCs and hBMMSCs. (H-L) Pluripotency assay of hBMMSC-iPSCs. EBs was formed (H) and spontaneously differentiated into many types of cells during the *in vitro* potential assay (I). (J-L) Hematoxylin and Eosin staining of teratoma sections from hBMMSC-iPSCs. 1×10^7^ of hBMMSC-iPSCs were injected subcutaneously into limbs of NOD/SCID mice. Teratomas were obtained after 8–10 weeks. Three germ layer as ectoderm, neural-like tissue (J); mesoderm, adipose tissue (K) and endoderm, intestinal-like epithelium (L) were observed.

We cultured hBMMSCs to passage 2–4 before reprogramming them into iPSC. cDNAs encoding four transcription factors were retrovirally transduced to hBMMSCs for iPSC generation. ESC-like colonies were examined with standard characterization procedures. AP staining showed that some ESC-like colonies (Fig. 1A) exhibited comparatively strong AP activity (Fig. 1B), whereas hBMMSCs had weak AP activity. No ESC-like colonies were observed in hBMMSC cultures. Immunofluorescence assay revealed that the colonies expressed pluripotent markers such as OCT4 (Fig. 1C), NANOG (Fig. 1D), SSEA3 (Fig. 1E), and TRA-1-81 (Fig. 1F). RT-PCR analysis verified the expression of pluripotent genes such as Nanog, Oct4, Sox2, and Rex1 (Fig. 1G). The differentiation potential of the colonies was determined with spontaneous differentiation *in vitro* (Figs. 1H-I) and teratoma formation *in vivo* (Figs. 1J–L). Taken together, the colonies had features typical of ESC colonies in morphology, expression of specific markers of pluripotency, and differentiation potential, indicating that iPSCs had been generated from hBMMSCs.

iPSCs derived from human skin fibroblasts (hFib-iPSCs) were used for parallel analysis. Because of the highly similar results, data for the generation and characterization of hFib-iPSCs are not shown here.

### Efficient commitment of hBMMSC-iPSCs into CD34+ progenitor cells by stepwise treatment with defined factors

**Figure 2:**
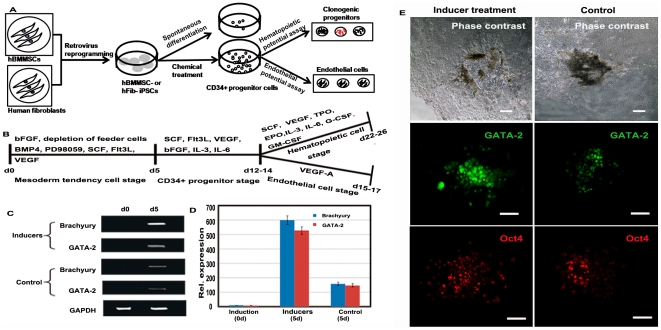
Exploration the potential of hBMMSC-iPSCs into CD34+ progenitor cells with the defined factors. (A) Schematic of CD34+ progenitor cell differentiation protocol for the study. (B) Protocol for the chosen defined factors, divided into groups according to their function, to direct hBMMSC-iPSCs to CD34+ progenitor cells, and then to hematopoietic and endothelial cells. (C-D) Enhanced expression of mesoderm transcription factor Brachyury and GATA-2 was detected in factor-treated hBMMSC-iPSCs, compared to the spontaneous differentiation groups by RT-PCR (C) and fluorescence intensity (D) assay after the differentiation for 5 days. The values were the mean ± SD of 3 independent experiments. (E) Immunoassay revealed that the hematopoietic transcription factor GATA-2 was up-regulated after factor treatment for 5 days and it was more efficient in hBMMSC-iPSC groups treated with the factors than in the spontaneous groups (control). The expression of the plutipotent marker Oct4 was similar between two groups. Scale bar, 100 µm.

In this study, we followed a defined culture condition protocol for CD34+ progenitor cell differentiation from hBMMSC-iPSCs. We did so to evaluate the potential of iPSCs for therapeutic applications. The culture scheme is given in Figure 2A. Factors representing essential lineage-inducing factors for mesodermal, hematopoietic, and endothelial cells, such as BMP4, PD98059, Flt3L, SCF, and VEGF, were selected and divided into groups by function, as shown in Figure 2B. In the initial induction step, the culture for hBMMSC-iPSCs were depleted of the feeder layer and bFGF, with the presence of BMP4, PD98059, Flt3L, SCF, and VEGF for 5 days to induce cell aggregate formation. The results of RT-PCR and immunofluorescence assays showed that Brachyury and GATA-2 were up-regulated at this time. Higher expression levels were observed in the groups treated with the inducer cocktail than in the spontaneous differentiation groups (Figs. 2C-D). This indicates that the factors employed here increased the potential of hBMMSC-iPSC commitment to mesoderm cells. Immunofluorescence assays confirmed that the transcription factor GATA-2 was up-regulated and that efficiency was more pronounced in hBMMSC-iPSC groups treated with the cocktail than in the spontaneous differentiation groups. However, the expression of the pluripotent marker OCT4 was similar in both groups (Fig. 2E).

**Figure 3:**
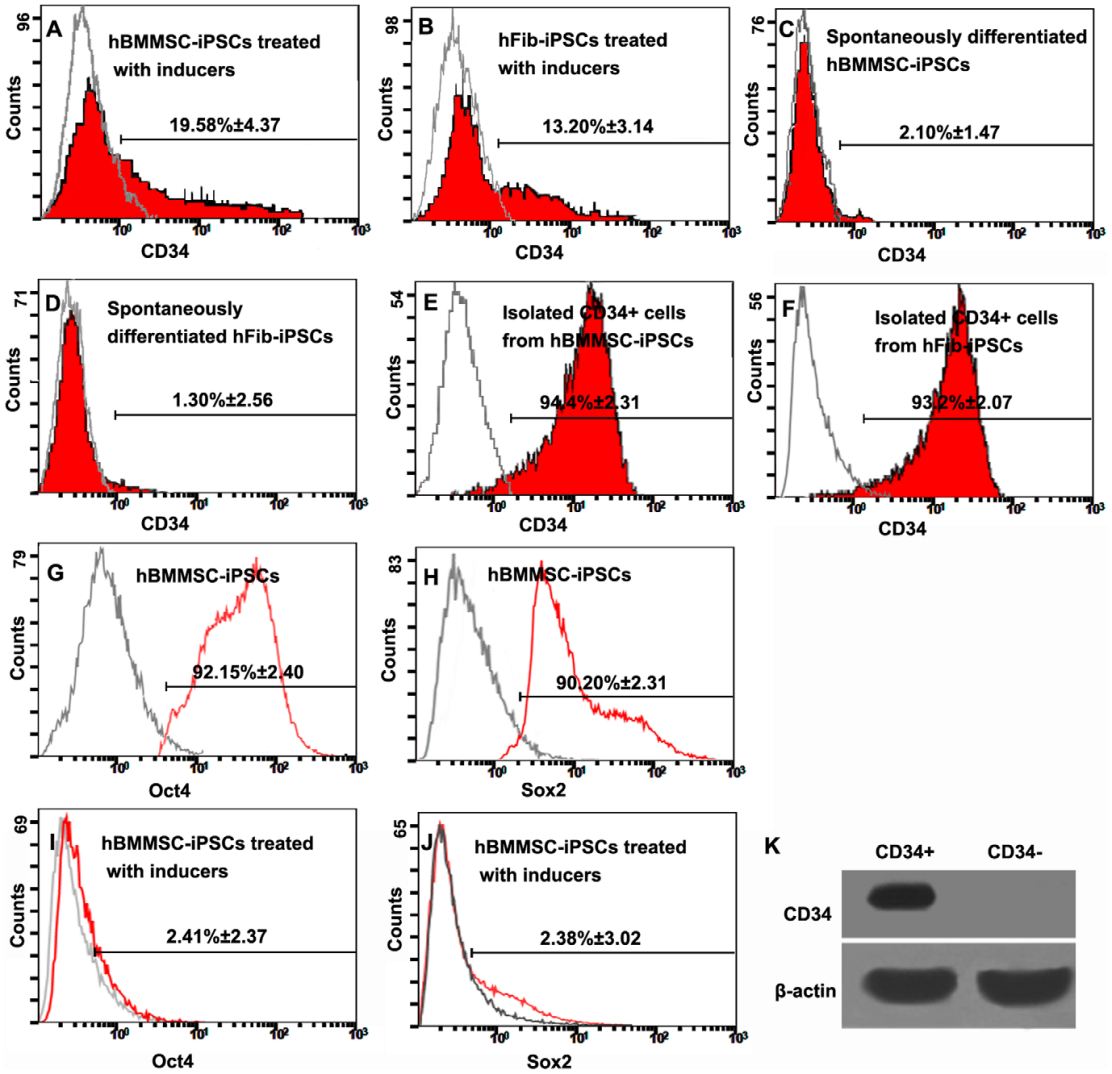
Efficient commitment of hBMMSC-iPSCs into CD34+ progenitor cells by treatment with the defined factors. CD34+ progenitor cells were identified by flow cytometry analysis in the groups. Differentiated hBMMSC-iPSCs treated with the factors produced about 20% proportion of CD34+ progenitor cells (A), higher than that of nearly 13% from hFib-iPSCs by parallel performance (B). However, the efficiency for the spontaneous differentiation groups was low. The proportion in hBMMSC-iPSCs treated with no factor was nearly 2% (C) and it was much lower in hFib-iPSCs of nearly 1% (D). The data demonstrated that the defined factors employed here efficiently improved the efficient differentiation of CD34+ progenitor cells from iPSCs, and iPSCs derived from hBMMSCs was more efficient to produce CD34+ progenitor cells than those derived from human fibroblasts. The enriched CD34+ progenitor cells were analyzed from hBMMSC-iPSC versus hFib-iPSC differentiation systems by flow cytometry (E-F). (G-J) Flow cytometry analysis demonstrated that the expression of Oct4 (G and I) and Sox2 (H and J) was down regulated at day 14 after the differentiation. (K) The chemically induced cells were separated into CD34+ and CD34- subsets and the former only had detectable CD34 transcripts.

After treatment for another 7–9 days with the cocktail containing SCF, Flt3L, VEGF, bFGF, IL-3, and IL-6, the mixed population displayed a series of changes. CD34+ progenitor cells, undetectable in undifferentiated hBMMSC-iPSCs, increased in number, as assessed by flow cytometry analysis. The proportion reached 19.58±4.37% (mean ± SD, n = 6, Fig. 3A), higher than that observed in parallel hFib-iPSCs (13.20±3.14%, mean ± SD, n = 6, Fig. 3B), about 10 fold that observed in spontaneously differentiated hBMMSC-iPSCs (2.10±1.47%, mean ± SD, n = 4, Fig. 3C), and about 20 fold that of spontaneously differentiated hFib-iPSCs (1.30±2.56%, mean ± SD, n = 4, Fig. 3D) (*P*<0.05). CD34+ progenitor cells were then isolated and enriched from the induced population by magnetic procedure and the purity reached more than 90% (Figs. 3E-F). The different replicates were derived from independent reprogramming cells. Differentiation of hBMMSC-iPSCs was confirmed by the down expression of pluripotency markers, Oct4 and Sox2 by flow cytometry, as shown in Figures 3G-J.

Additionally, we separated the chemically induced cells into CD34+ and CD34- subsets and demonstrated that the former only had detectable CD34 transcripts (Fig. 3K).

### Dynamic analysis during hematopoietic cell differentiation of hBMMSC-iPSCs

**Figure 4:**
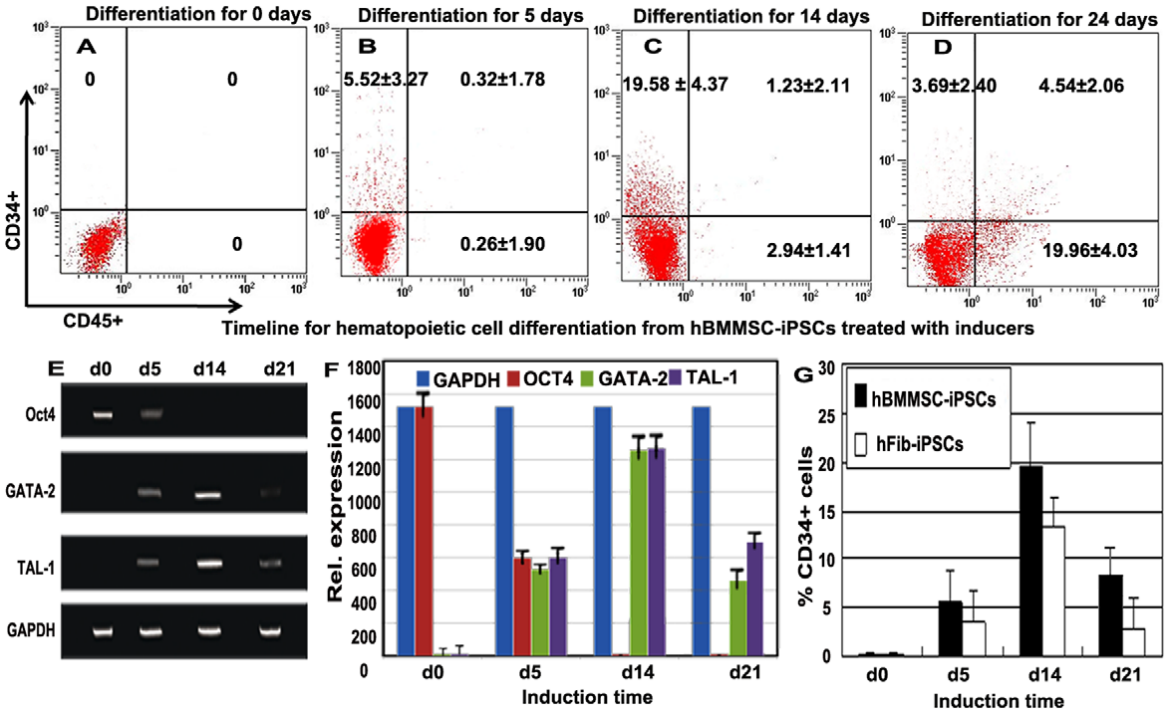
Kinetic expression of the hematopoietic and pluripotent genes during hBMMSC-iPSC commitment to CD34+ progenitor cells, and then to hematopoietic cells. (A-D) Kinetic expression of CD34+ and CD45+ during the hematopoietic cell differentiation of hBMMSC-iPSCs with flow cytometry analysis. Undifferentiated hBMMSC-iPSCs expressed no CD34 or CD45 (A). After treatment with the cocktail containing mesodermal, hematopoietic, and endothelial inducers for 5 days, hBMMSC-iPSCs expressed about 5% percentage of CD34+ but few CD45+ cells (B). After culturing with the following hematopoietic and endothelial inducer cocktail for additional 7–9 days, the proportion of CD34+ population increased to nearly 20% and a few CD45+ cells were obtained at this stage (C). In hematopoietic potential assay of CD34+ progenitor cells, about 5% population of CD34+CD45+ cells were developed from the CFU assay, and the percentage of CD45+ cells increased to about 25% during the differentiation culture (D). (E-F) Dynamically relative expression of the pluripotent marker Oct4 and the hematopoietic cell markers TAL-1, SCL during hBMMSC-iPSC commitment to CD34+ progenitor cells, and then to hematopoietic cells by RT-PCR (E) and fluorescence intensity (F) assay in the differentiation culture. The values were the mean ± SD of 3 independent experiments. (G) Kinetics of CD34+ cells during hematopoietic differentiation of hBMMSC-iPSCs versus hFib-iPSCs treated with the inducers.

Flow cytometry and RT-PCR assays were used to detect the dynamic course of hematopoietic differentiation of hBMMSC-iPSCs via treatment with the cocktails. The results of flow cytometry analysis were shown in figure 4A-D. Undifferentiated hBMMSC-iPSCs expressed no CD34 or CD45 (Fig. 4A), and a very low percentage of CD34+ cells, but no CD45+ cells were detected after induction for 5 days (Fig. 4B). After treatment with the following cocktail for another 7–9 days, the number of CD34+ increased significantly relative to the total number of cells. However, few CD45+ cells were obtained at this stage (Fig. 4C). As the hematopoietic induction continued, samples obtained from the CFU assay developed a subpopulation of CD34+CD45+ cells, and the percentage increased until about 24 days of culture (Fig. 4D). This marked increase in CD45+ cells reflects the development of more mature hematopoietic cells. The result of the hematologic differentiation of hBMMSC-iPSCs shown here suggests that the hematopoiesis was a dynamic process. RT-PCR and Image J analysis demonstrated that the pluripotent marker Oct4 continued to be down-regulated and the hematopoietic special genes TAL-1 and SCL were sustainably up-regulated during the process (Figs. 4E-F). At day 14 after the differentiation, the expression of TAL-1 and SCL reached a considerable level while the expression of Oct4 was not detected (Figs. 4E-F). The kinetics of CD34+ cells from hBMMSC-iPSCs versus hFib-iPSCs treated with the cocktails was also detected as shown in figure 4G, indicating that the efficiency of hematopoietic differentiation from hBMMSC-iPSCs was more efficient than that from hFib-iPSCs.

### Differentiation of hBMMSC-iPSC-derived CD34+ progenitor cells into hematopoietic cell lineages

**Figure 5:**
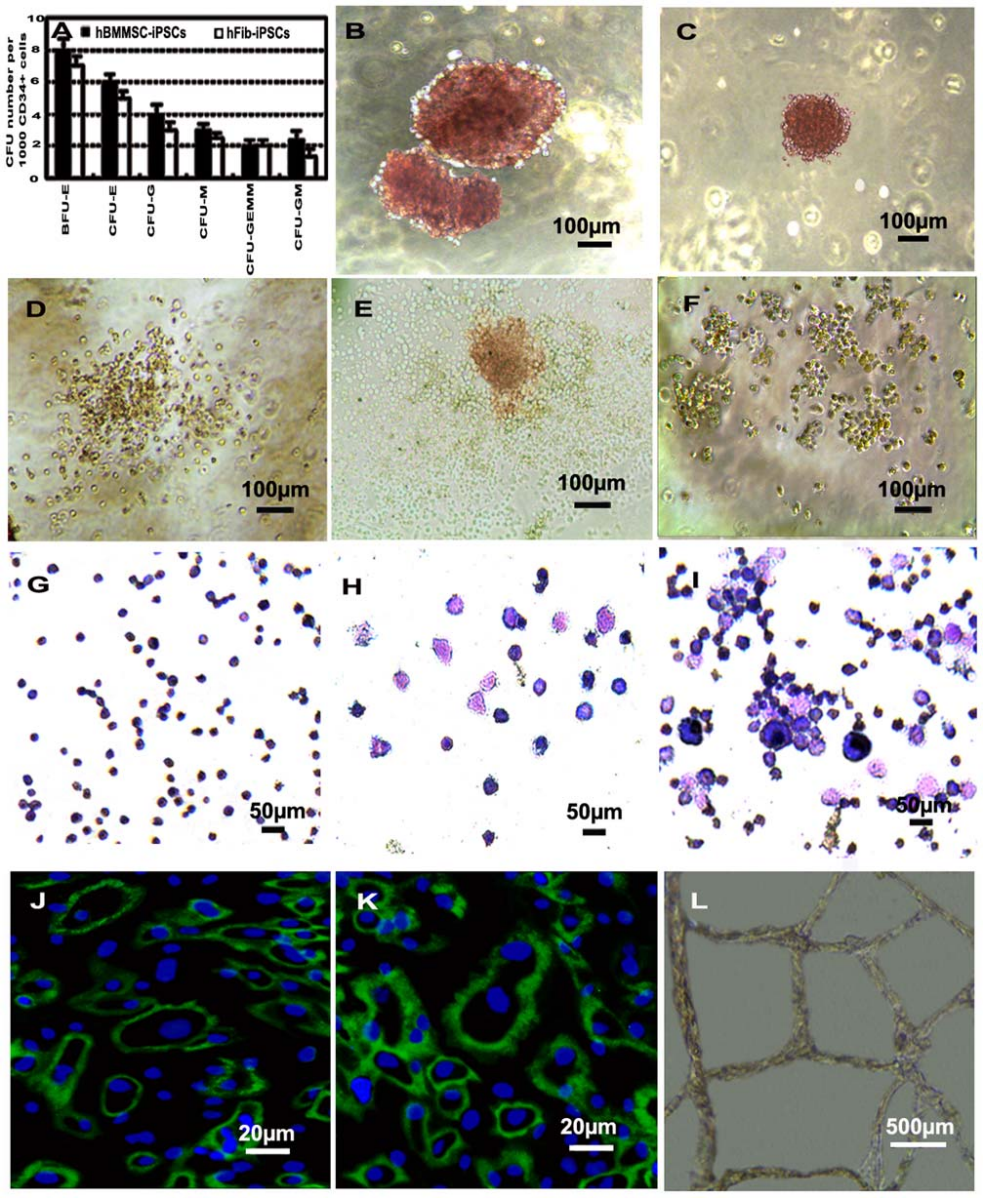
Hematopoietic and endothelial cell potential assay of CD34+ progenitor cells obtained from differentiated hBMMSC-iPSCs. (A-I) Hematopoietic CFU formation and hematopoietic lineages assay for the potential of CD34+ progenitor cells. (A) Measurement for CFU potential of CD34+ cells derived from hBMMSC-iPSCs compared to that from hFib-iPSCs. (B-F) Various types of CFUs were observed with a reversed telescope as BFU-E (B), CFU-E (C), CFU-GM (D), CFU-GEMM (E) and CFU-G (F) when CD34+ progenitor cells derived from hBMMSC-iPSCs were seeded into CFU forming culture system to assess the short-term differentiation capabilities of hematopoietic progenitors. (G-I) Morphologies of the hematopoietic lineages derived from CFU culture of CD34+ progenitor cell commitment to hematopoietic cells observed with a reversed telescope (Olympus), including erythroid from BFU-E (G), granulocyte from CFU-G (H) and macrophage, granulocyte, erythroid, megakaryocyte from CFU-GEMM (I). (J-L) Analysis of endothelial cell potential of CD34+ progenitor cells derived from hBMMSC-iPSCs. Some of CD34+ progenitor cells cultured with EGM-2 were positive to CD31 (J) and VE-CADHERIN (K). When treated with VEGF-A on Matrigel for 3 days, vascular-like structures were photographed (L).

CD34+ progenitor cells were isolated to determine their CFU potential in methylcellulose and in hematopoietic cytokines including SCF, VEGF, TPO, EPO, IL-3, IL-6, and GM-CSF. CFUs were enumerated 14 days later and the number of colonies in the hBMMSC-iPSC groups is higher than that in the hFib-iPSC groups (Fig. 5A).

**Figure 6:**
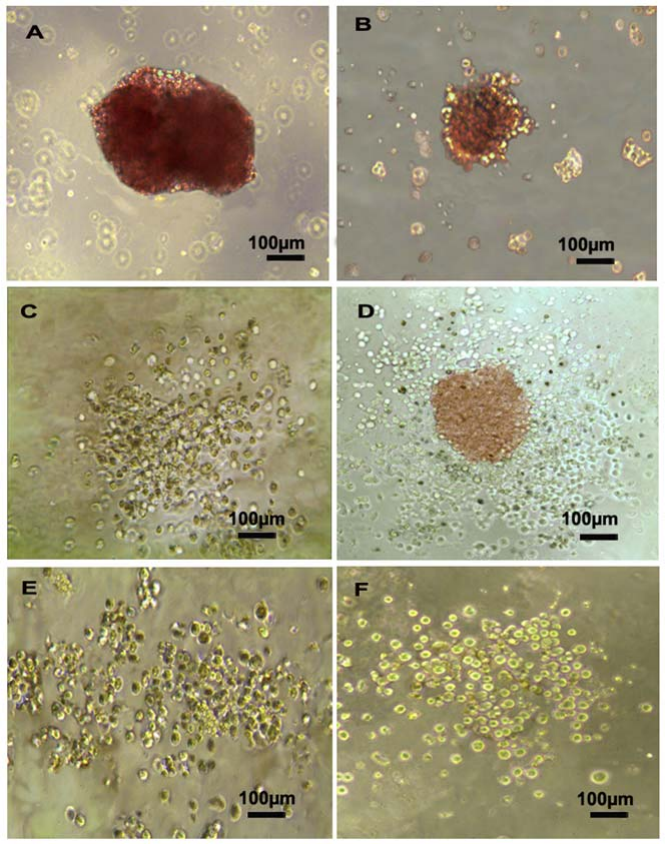
Representative colony morphologies from hFib-iPSC-derived CD34+ cells. CD34+ progenitor cells obtained from hFib-iPSCs were cultured in CFU forming culture system for hematopoietic CFU formation. Various types of CFUs were observed as BFU-E (A), CFU-E (B), CFU-GM (C), CFU-GEMM (D), CFU-G (E), and CFU-M (F).

Various CFUs were identified in the culture after 10–14 days. These CFUs included burst-forming units of erythroid lineages (BFU-E) (Fig. 5B), small colonies of erythroid cells (CFU-E) (Fig. 5C), CFU-granulocytes and macrophages (CFU-GM) (Fig. 5D), CFU-granulocytes, erythroid cells, macrophages, and megakaryocytes (CFU-GEMM) (Fig. 5E), and CFU-granulocytes (CFU-G) (Fig. 5F). Hematopoietic cell lineages including erythroid cells, granulocytes, megakaryocytes, and macrophages were characterized using Wright-Giemsa staining (Figs. 5G–I). Additionally, representative colony morphologies including BFU-E (Fig. 6A), CFU-E (Fig. 6B), CFU-GM (Fig. 6C), CFU-GEMM (Fig. 6D), CFU-G (Fig. 6E), and CFU-M (Fig. 6F) from hFib-iPSC-derived CD34+ cells were shown as control.

### Differentiation of hBMMSC-iPSC-derived CD34+ progenitor cells into endothelial cells

In this study, hBMMSC-iPSC-derived CD34+ progenitor cells were differentiated into endothelial cells in endothelial cell growth medium-2 (EGM-2). Immunoassay results showed that some hBMMSC-iPSC-derived CD34+ progenitor cells could differentiate into endothelial cells, as determined by the expression of endothelial cell-specific proteins, including CD31 and VE-CADHERIN (Figs. 5J and 5K). These cells formed vascular-like structures on Matrigel when the cultures were supplemented with VEGF-A (Fig. 5L).

## Discussion

Human somatic cells can be reprogrammed into a pluripotent state by ectopic expression of pluripotent transcription factors. This process provides a new source of cells with the capacity of indefinite proliferation and patient-matched HLA specificity suitable for cell-based therapy, eliminating the risk of GVHD and the need for immunosuppressive drugs or immunomodulatory protocols in cell transplantation [14]–[17].

Efficient commitment to functional CD34+ progenitor cells from human iPSC is crucial for the clinical application of iPSC. Although previous studies have demonstrated that pluripotent cells can be differentiated into hematopoietic cells using either stromal cell co-culture or EB–based systems [9], [23]–[25], [29], the induction efficacy and cellular function of CD34+ progenitor cells from ESCs or iPSCs vary greatly with the different protocols, due to differences in cell derivation methods, cytokine composition, and types of feeder support. In the present study, the spontaneous differentiation approach involving EB formation was found to be inefficient. Feeder support systems were precluded because of the high risk of contamination from unidentified factors [18], [19]. Several other studies have reported that hematopoietic progenitors can be generated from iPSCs in fully defined conditions [20], [21]. However, these reports note that overall efficacy remains low and hiPSC-derived CD34+ cells cannot form hematopoietic colonies [21].

In this study, we selected hBMMSC-derived iPSCs to produce CD34+ progenitor cells because BMMSC have several advantages over other cell types: they provide an environment for hematopoiesis, their epigenetic state might favor hematopoiesis, and they have been widely used in clinics for many years. Meanwhile, we selected the defined factors that can specifically promote the functions of mesodermal, hematological, and endothelial cells, including BMP4, SCF, Flt3L, VEGF, IL-3, etc. We then used these factors to induce the differentiation of hBMMSC-iPSC into CD34+ progenitor cells. Factors that can affect mesoderm signaling pathways, such as BMP4 and PD98059, were used to enhance mesoderm differentiation and to initiate the production of CD34+ progenitor cells [20], [21], [23], [30]. Flt3L, SCF, and VEGF are early-acting hematopoietic and endothelial cell factors [20], [21], [30]–[34]. They were used here to activate early hematologic and endothelial signaling pathways in the differentiation system. After iPSCs were cultured for 5 days in the differentiation medium, a higher percentage of mesoderm cells were induced in the presence of the first inducer cocktail, including BMP4 and SCF, than without the cocktail supplement (*P*<0.05). These mesoderm cells were then induced to become CD34+ progenitor cells by culturing with the second cocktail, which include SCF and IL-3, for another 7–9 days. Cellular morphology and the expression of the hematopoietic transcription factors TAL-1 and SCL in these cells confirmed that they are hematopoietic cells. The differentiated hBMMSC-iPSCs contained nearly 20% of CD34+ progenitor cells, more than that from hFib-iPSCs (nearly 13%) or the spontaneous differentiation groups (*P*<0.05). These results demonstrate that the factors employed here could efficiently differentiate iPSCs into CD34+ progenitor cells, and CD34+ progenitor cells could be more efficiently generated from hBMMSC-iPSCs than from hFib-iPSCs.

We further verified that the CD34+ cell population had hematopoietic and endothelial cell potential. RT-PCR showed that the differentiation population expressed hematopoietic transcription factors TAL-1 and SCL. When these cells were subjected to the hematopoietic colony-forming cell assay, various hematopoietic CFU colonies were formed. These included CFU-E, BFU-E, CFU-G, CFU-GM, and CFU-GEMM, indicating that these CD34+ cells are truly multipotent, with the ability to form all types of blood cells. The number of CFUs derived from hBMMSC-iPSC groups was greater than that from hFib-iPSCs. One possible explanation is that hBMMSC-iPSCs might retain their epigenetic memory. Furthermore, the time for CFUs identification was various [35]–[37], and the reason may be that the cells and the culture system are different. In our study, we observed BFU-E and CFU-E forming with small size during the first week and the colonies would grow following the next days. We identified various CFUs in the culture during 10–14 days and enumerated CFUs at day 14. CD34+ progenitor cells were also found to have endothelial potential. When cultured in endothelial cell medium, these cells produced CD31 and VE-CADHERIN, which were endothelial specific adhesion molecules. Formation of vascular-like structures in matrigels further confirmed their endothelial identity.

During the hematopoietic commitment of hBMMSC-iPSCs, the pluripotent gene Oct4 continued to be down-regulated and its expression completely vanished at day 14 after induction. However, the expression of the hematologic transcription factors TAL-1 and SCL underwent a dynamic process, showing up-regulation at the initial induction stage and then gradual down-regulation after CD34+ progenitor cell commitment to CFU formation. The down-regulation and disappearance of Oct4 expression seems contradictory to the reports of direct conversion of human fibroblasts to multilineage blood progenitors by ectopic expression of Oct4 [38]. One explanation is that Oct4 activation of hematopoietic transcription factors in only a specific stage of early hematopoiesis. Another explanation is that other POU domain proteins, such as OCT1 and OCT2, might have a redundant role in hematopoietic fate conversion [38]. However, the mechanism merits further study.

Seiler *et al.* reported that compare to fibroblast-derived iPSCs, iPSCs generated from mouse bone marrow hematopoietic progenitor cells have severely reduced capacity to differentiate from mesodermal to hematopoietic progenitor cells [39]. The authors found that elevated expression of the ectopic transcription factors Sox-2, Oct-4 and Klf4 inhibited the differentiation of iPSC to hematopoietic progenitor cells. To generate iPSCs, they used the pMY vectors, which retrovirally transduced the cells and caused irreversible ectopic expression of the transduced genes. To circumvent this problem, we used the pMXs-based retroviral vectors, and at day 14 after the differentiation, we could no longer detect the expression of Sox-2 and Oct-4.

In conclusion, CD34+ progenitor cells, retaining hematopoietic and endothelial cell potential, can be efficiently induced from hBMMSC-iPSCs with a two-step, fully defined culture system. Additionally, hBMMSC-derived iPSCs were found to produce CD34+ progenitor cells more efficiently than human-skin-fibroblast-derived iPSCs did. This system provides a novel method of generating personalized CD34+ progenitor cells, which can potentially be used for cellular therapy, drug screening, and hematologic disease study. However, to determine the real biology and safety of iPSC-derived hematopoietic cells, their *in vivo* function should be carried out in the future. There might be a long way to go for reprogrammed cell therapeutic use.

## Materials and Methods

### Cell culture

hBMMSCs were used as target cells for reprogramming. hBMMSCs were recovered from liquid nitrogen which were frozen by our team members and saved in our laboratory. The characteristics of these cells have been identified [40]. hBMMSCs were cultured in a flask at a density of 10^7^ cells per 75 cm^2^ in DMEM (GIBCO) supplemented with 10% fetal bovine serum (FBS) (GIBCO), 100 U/ml ampicillin, and 100 U/ml streptomycin (GIBCO) at 37°C in 5% CO_2_ atmosphere. hBMMSCs were split with 0.25% trypsin/ethylene diaminetetraacetic acid (trypsin-EDTA, Invitrogen) when cells reached 90% confluence. hBMMSCs at passages 2–4 were used for iPSC generation.

### Generation of iPSCs from hBMMSCs

Retroviral supernatants were prepared as described by Yamanaka S *et al.* with some modifications [16]. Briefly, each pMXs-based retroviral vector containing reprogramming factors –hOct4, –hSox2, –hcMyc, and –hKlf4 (Addgene: http://www.addgene.org) was used with a mixture of two helper plasmids: pCMV-G and pCMV-GP. Two micromoles of VPA (Sigma) and 25 µg/ml Vc (Sigma) were supplemented to improve efficiency. Cultures were maintained at 37°C, 5% CO_2_ with daily medium changes. VPA was supplemented for 7 days and Vc was added until colonies were picked for expansion. During the reprogramming, colonies were observed daily with a reversed microscope (Olympus). Based on cell morphology at 15–18 days after the initial infection, colonies were picked for expansion culture on mitomycin-C-treated mouse embryonic fibroblasts (CF1, SiDanSai Biotechnology Co. , Ltd, Shanghai, China) in hESC culture medium consisting of 80% DMEM/F12 (Invitrogen), 20% knockout serum replacement (KSR) (Invitrogen), 1 mM L-glutamine (Invitrogen), 1% non-essential amino acids (NEAA) (Invitrogen), 0.1 mM β-mercaptoethanol (Invitrogen), and 4 ng/ml basic fibroblast growth factor (bFGF) (Invitrogen). iPSCs were passaged by 1 mg/ml collagenase IV (Invitrogen). The medium was changed every day. Colonies expanded up to 10 passages were used for determination of iPSC characteristics. Alkaline phosphatase (AP) staining was performed with an alkaline phosphatase detection kit (Millipore) according to the manufacturer's instructions. Differentiation potential *in vitro* and *in vivo* analysis was carried out as described by Yu *et al*. [15].

Human-skin-fibroblast-derived iPSCs (hFib-iPSCs) served as control groups in the assessment of the efficiency of the CD34+ progenitor cells under the same treatment conditions. hFib-iPSCs were generated from human skin fibroblasts using the same protocol, given above.

### Immunofluorescence assays

For analysis of immunofluorescence, cells were fixed with 4% paraformaldehyde at room temperature for 15 minutes. When nuclear proteins were detected, cells were permeabilized with 0.1% Triton X-100 in PBS and then blocked with normal goat serum for 1 hour at room temperature. Staining was performed using the following antibodies (Santa Cruz): anti-OCT4, anti-NANOG, anti-SSEA3, anti-TRA-1-81, anti-GATA-2, anti-CD31, and anti-VE-CADHERIN overnight at 4°C. Then the cells were washed several times with PBST (0.1% Tween-20 in PBS). Secondary antibodies conjugated to FITC or Cy3 (Boster) were used for fluorescence detection followed by counterstaining with DAPI (Boster) to reveal nuclei. Images were obtained using a confocal microscope (LSM 510 Meta, Zeiss).

### RT-PCR analysis

The primer sets for reverse transcription PCR analysis of endogenous pluripotent genes and hematopoietic commitment gene expression were designed as shown in Table 1 using Primer Premier 5 software. Total RNA was prepared using TRIzol (Invitrogen) according to the manufacturer's protocol, and then used for cDNA synthesis. First-strand cDNA was synthesized with Superscript III First Strand Synthesis SuperMix (Invitrogen) for quantitative reverse transcription PCR. GAPDH was used as an endogenous control. PCR was performed in a 20 µl mixture containing 1× PCR buffer, 0.5 U of Taq DNA polymerase, 0.2 mM each of dNTP and 1.5 mM MgCl2, 0.2 µM of each primer, and 2 µl of each RT product as a template. The following program was carried out: initial denaturation for 4 min at 94°C, 35 cycles of 94°C for 15 s, then 35 s at temperatures specific to each transcript (51°C for Nanog and Rex-1; 53°C for GAPDH, Brachyury, and TAL-1; 55°C for Oct4; 58°C for GATA-2; 60°C for SCL ; 68°C for Sox2), and 72°C for 1 min. This was followed by 72°C for 10 min. cDNA from hBMMSCs was used as a negative control. cDNA from human ESC line H1 served as a positive control. Image J software was used to measure the intensity to determine the influence of chemical treatment on hBMMSC-iPSC commitment to CD34+ progenitor cells. The reactions were measured in triplicate.

**Table:** **Table 1.** Primer sets for the pluripotent genes, hematopoietic commitment genes by RT-PCR.

Genes	Sequence (5′ to 3′) F	Sequence (5′ to 3′) R
Oct4	TTCAGCCAAACGACCATC	GGAAAGGGACCGAGGAGTA
Sox2	AAACAGCCCGGACCGCGTCAA	TCGCAGCCGCTTAGCCTCGT
Nanog	CCTATGCCTGTGATTTG	AGAAGTGGGTTGTTTGC
Rex1	GGCAAAGACAAGACACC	GCAAATTCTGCGAGCT
GAPDH	AAGGTCGGAGTCAACGG	GGAAGATGGTGATGGGATT
Brachyury	TGAGCCTCGAATCCACAT	GGGCACCTCCAAACTGA
GATA-2	GGCGTCAAGTACCAGGTGT	GGTCGGTTCTGCCCATTC
SCL	ATGCCTTCCCTATGTTCACCA CCA	TGAAGATAGCCGCACAACTT TGG
TAL-1	TTGTGCGGCGTATCTTC	CAGGGTCCTTGCCAGTC

### EB formation of hBMMSC-iPSCs by defined chemicals and growth factors

At confluence, undifferentiated iPSCs were treated with collagenase IV and then cultured for cell aggregate formation with the feeder layer and bFGF depleted from the culture system. The cells were fed with the differentiation medium (KnockOut DMEM, 2 mM GlutaMAX,1% NEAA, 0.1 mM β-mercaptoethanol, 50 U/mL penicillin, 50 mg/mL streptomycin) and the first group of the defined factors, including 20 ng/mL bone morphogenetic protein 4 (BMP4), 50 µM PD98059, 300 ng/mL stem cell factor (SCF), 300 ng/mL FMS-like tyrosine kinase 3 ligand (Flt3L) (all from R&D Systems), and 100 ng/mL vascular endothelial growth factor (VEGF) (StemCell). The spontaneous differentiation cultures treated with differentiation medium alone served as controls. After about 5 days of culture, the populations of small embryoid bodies were generated and harvested. Some of the embryoid bodies were collected to determine the expression of the pluripotent marker OCT4 and the mesodermal transcription factors Brachyury and GATA-2 by immunofluorescence and RT-PCR assays. The rest of the cells were prepared for CD34+ progenitor cell commitment using the following growth factor cocktail treatment or by allowing cells to spontaneously differentiate into various types of cells representing the endoderm, ectoderm, and mesoderm.

### CD34+ progenitor cell differentiation by growth factor treatment

For CD34+ progenitor cell differentiation, hBMMSC-iPSCs were cultured with the differentiation medium supplemented with BMP4, PD98059, SCF, Flt3L, and VEGF for 5 days. They were then dissociated into single-cell suspensions using collegenase IV followed by 0.25% trypsin-EDTA and plated on low-cluster plates in StemPro-34 (StemCell) supplemented with the second set of growth factors: 300 ng/mL SCF, 300 ng/mL Flt3L, 100 ng/mL VEGF, 5 ng/mL bFGF, 20 ng/mL interleukin-3 (IL-3) (StemCell) and 20 ng/mL interleukin-6 (IL-6) (StemCell) for an additional 7–9 days to induce CD34+ progenitor cell formation. The cells were then prepared for further characterization by flow cytometry analysis for CD34+ progenitor cells and CD45+ cells and by RT-PCR for the expression of the pluripotent marker OCT4 and hematopoietic transcription factors TAL-1 and SCL. Several other assays were performed to demonstrate induced cell commitment to hematopoietic and endothelial cells.

Regarding assessment of the efficiency of iPSC spontaneous differentiation to CD34+ progenitor cells, EBs obtained from the differentiation medium alone were cultured on low-cluster plates in StemPro-34 supplemented with no chemicals for an additional 7–9 days, and then the cells were prepared for analysis of CD34+ progenitor cells by flow cytometry.

### Flow cytometry analysis

Flow cytometry analysis was performed as described by Thomson with some modifications [24]. Briefly, the differentiated cell mixtures were washed in Ca^2+^- and Mg^2+^-free PBS and dissociated with 1 mg/ml collagenase IV and 0.05% trypsin/0.53 mM EDTA supplemented with 1% mouse serum (GIBCO) for 5–10 min. Cell clumps were removed from the dissociated cells by filtering the mixture through 40 µm cell strainers. The single cell suspension (1×10^6^) was prepared in PBS-1% FBS containing 0.1% sodium azide, and labeled with either isotype control or antigen-specific antibodies. Directly conjugated isotype control antibodies IgG1-FITC and IgG1-phycoerythrin (PE) (BD) were used to determine the autofluorescence background of the cells. As for nuclear proteins of Oct4 and Sox2, the cells are fixed and treated with 0.1% Triton X-100 before staining. The following antibodies were used: CD34-PE, CD45-FITC, Oct4-FITC, and Sox2-FITC (all purchased from BD). Dead cells were stained with the seven-aminoactinomycin (7-AAD) staining solution (BD), and excluded from the analysis. The analysis of antibody-labeled cells was performed using a BD FACScalibur instrument (BD).The data were analyzed using FlowJo Version 7.2.5 software. Independent experiments for flow cytometry assays were performed in triplicate.

### CD34+ progenitor cell enrichment

CD34+ progenitor cells were collected using CD34 MicroBead Kit (Milteneyi Biotech) on MiniMACS device (Milteneyi Biotech) according to the manufacturer's instructions.

### Colony-forming unit assay

Colony-forming unit (CFU) assay was performed to analyze the hematopoietic colony-forming potential of CD34+ progenitor cells isolated from the differentiation system.

The cells were dissociated with 0.25% trypsin/EDTA, dispersed by passaging them through a 20-gauge needle, and then transferred at 5000 cells per ml onto a low adherent dish with IMDM containing 9% MethoCult supplemented with 0.1 mM 2-mercaptoethanol, 2 mM GlutaMAX, and the following cytokines: 100 ng/mL SCF, 100 ng/mL VEGF, 3 U/mL thrombopoietin (TPO), 3 U/mL erythropoietin (EPO), 20 ng/mL IL-3, 20 ng/mL IL-6, 50 ng/mL granulocyte colony-stimulating factor (G-CSF), and 50 ng/mL granulocyte-macrophage colony-stimulating factor (GM-CSF) (all from StemCell) for a further 12–14 days to promote hematopoietic specification. The progenitors formed hematopoietic colonies. CFUs were determined and enumerated at days 22 to 26 after initial iPSC differentiation by colony shape, cell size, and the extent of visible cell content. Lineage assignment was confirmed with Wright-Giemsa staining within the CFU colonies by morphological analysis.

### Western blot

Cell extracts were prepared using RIPA Lysis and Extraction Buffer (Thermo). The total protein concentration was analyzed with Bio-Rad protein assay (Biorad) per the manufacturer's instructions. Forty micrograms of total proteins were prepared to standard western blot procedure followed by immunodetection with an anti-human CD34 antibody (santa cruz). B-actin was used as an internal control.

### Endothelial cell induction and vascular tube-like structure formation

For the assay of endothelial cell potential, CD34+ progenitor cells were plated onto fibronectin-coated (Invitrogen) plates in endothelial cell growth medium EGM-2 (BD Falcon). To determine the expression of CD31 and VE-CADHERIN by immunofluorescence assay, the endothelial cultures were fixed in 4% paraformaldehyde, permeabilized with 0.1% Triton X-100, and blocked with goat serum. Samples were labeled with mouse anti-human anti-CD31 (Santa Cruz) or mouse anti-human anti-VE-CADHERIN (Santa Cruz) followed by treatment with FITC-conjugated anti-mouse-IgG (Boster). They were then examined using a confocal microscope (LSM 510 Meta, Zeiss).

For the assay of the formation of vascular tube-like structures, 5×10^5^ CD34+ progenitor cells in a 6-well plate, derived from the differentiation system were incubated on Matrigel matrix (BD Biosciences) and solidified for 1 hour at 37°C in EGM-2 supplemented with 40 ng/ml vascular endothelial growth factor-A (VEGF-A) for 3 days. The vascular tube-like structures were photographed with an inverted microscope (Nikon).

### Statistical analysis

Statistical analyses were performed with SPSS software (version 13.0). *P* values less than 0.05 were accepted as statistically significant.
