# Correction: Efficient Commitment to Functional CD34+ Progenitor Cells from Human Bone Marrow Mesenchymal Stem-Cell-Derived Induced Pluripotent Stem Cells

**DOI:** 10.1371/annotation/ebddb7ce-712c-4f9d-8412-20dc827c30b9

**Published:** 2012-08-16

**Authors:** Yulin Xu, Lizhen Liu, Lifei Zhang, Shan Fu, Yongxian Hu, Yingjia Wang, Huarui Fu, Kangni Wu, Haowen Xiao, Senquan Liu, Xiaohong Yu, Weiyan Zheng, Bo Feng, He Huang

There were errors in the published text. SCL should be changed to GATA-2 in the Abstract, in the "Dynamic analysis during hematopoietic cell differentiation of hBMMSC-iPSCs" section of Results, in the Discussion, and in Materials and Methods. The primer sets for SCL should also be removed from Table 1 and "60°C for SCL" should be removed from the "RT-PCR analysis" section of Materials and Methods.

